# ‘Soccer toe’: Chronic physeal injury of the great toe metatarsal in a skeletally immature child – A case report

**DOI:** 10.4102/sajr.v24i1.1834

**Published:** 2020-04-22

**Authors:** Andrew Schapiro, Tal Laor

**Affiliations:** 1 Department of Radiology, University of Cincinnati, Cincinnati Children’s Hospital Medical Center, Cincinnati, OH, United States of America; 2 Department of Radiology, Harvard Medical School, Boston Children’s Hospital, Boston, MA, United States of America

**Keywords:** Physis, Growth plate, Toe, MRI, Stress, Musculoskeletal imaging, Sports medicine

## Abstract

Chronic physeal stress injuries in children can result from ongoing, repetitive compression, distraction and/or shear forces during sports-related activity, and manifest as physeal widening on imaging. We present an 11-year-old soccer athlete with focal physeal widening of her great toe metatarsal and postulate that ongoing or repetitive stress from soccer play may manifest as this imaging appearance. We suggest that recognition of this entity in growing children might explain pain, if present, and guide conservative treatment.

## Introduction

In its most recent worldwide survey, the Fédération Internationale de Football Association (FIFA) estimated that 265 million people play soccer worldwide, making it the world’s most popular sport. Although recognised as a relatively safe sport for children to play, soccer is nevertheless associated with a substantial injury toll.^1^ Most soccer-related musculoskeletal injuries in children are acute, but those that result from chronic overuse still account for 5% – 20% of the total and can result in weeks to months of missed playing time.^1,2^ Injuries because of chronic musculoskeletal overuse most commonly encountered in paediatric soccer players occur at the apophyses of the pelvis and lower extremities^1,2^ as a result of recurrent or ongoing traction at sites of tendon and muscle origin or insertion, and may manifest upon imaging as widening of apophyseal physes.^3^ Abnormal widening of the transverse physes of lower extremity long bones as a result of chronic compressive repetitive physeal stress injury has also been reported for the knee.^4^

We present a case report of an 11-year-old female soccer athlete who was imaged for lateral foot pain. She had no recognised or reported antecedent acute traumatic event to the great toe nor had any medial foot pain. On magnetic resonance (MR) imaging, there was focal dorsal widening of the great toe metatarsal physis in comparison to the unaffected portion of the physis and to the other physes in the same foot. We postulate that ongoing athletic activity that results in chronic physeal stress injury might manifest as dorsal physeal widening of the great toe metatarsal on MR imaging in a skeletally immature soccer player.

## Case report

An 11-year-old female soccer player presented to the orthopaedic clinic for acute lateral right ankle and foot pain after she rolled her ankle in a soccer game. She had no previous history of prior fracture in the foot, and apart from her current complaint, she was healthy. On physical examination, mild swelling with tenderness on palpation at the level of the lateral malleolus, inferior to the lateral malleolus, and along the lateral aspect of the foot was noted, but no pain was felt at the base of the great toe metatarsal. She had normal joint range of motion and no joint laxity. There were no associated skin changes.

**FIGURE 1:**
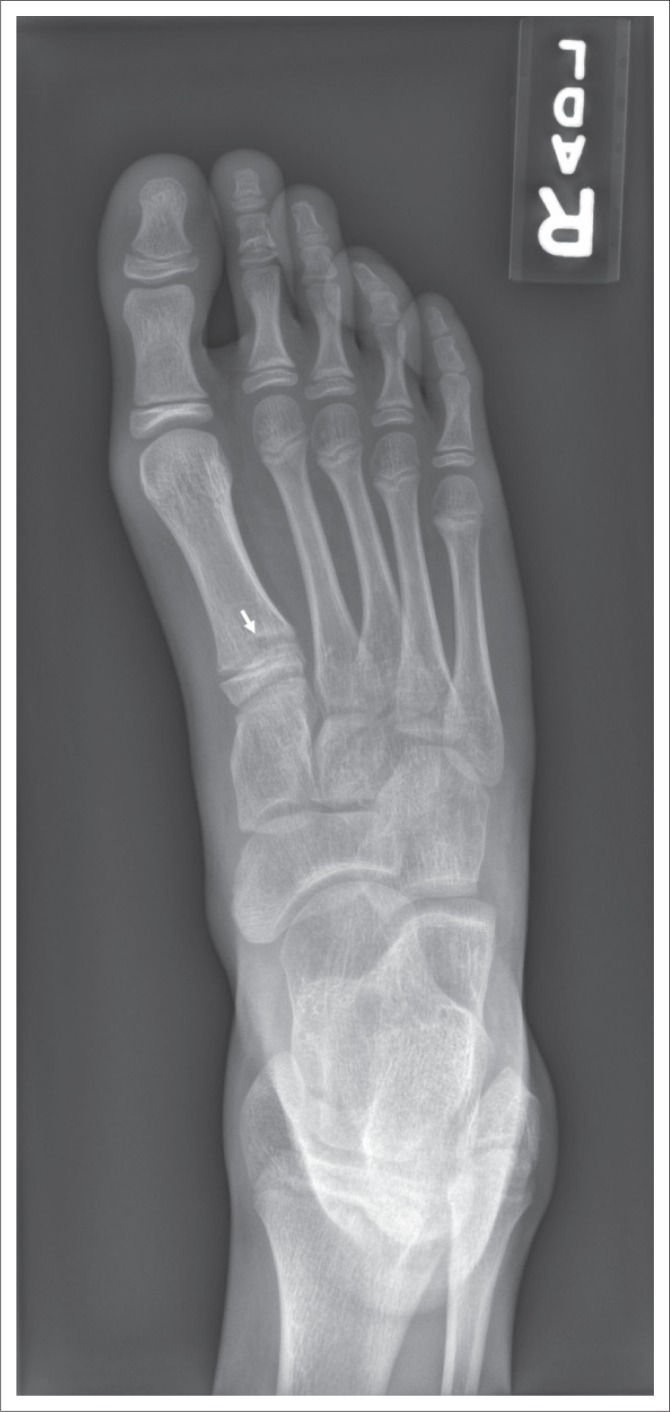
An 11-year-old female soccer player with lateral ankle and foot pain following a soccer injury. Frontal view of the foot shows subtle widening (arrow) of the physis of the great toe metatarsal. The remainder of the imaging examination is normal.

**FIGURE 2:**
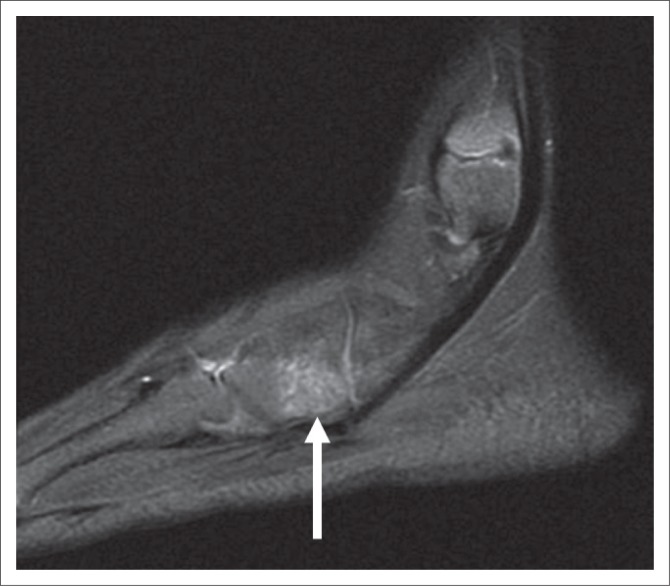
Cuboid contusion. Sagittal fast spin echo T2-weighted image (Repetition Time [TR]/Echo Time [TE] 3500/73 ms) of the lateral aspect of the foot shows focal oedema-like signal within the cuboid. No fracture line was evident. The finding presumably accounted for the patient’s lateral foot pain at presentation.

**FIGURE 3:**
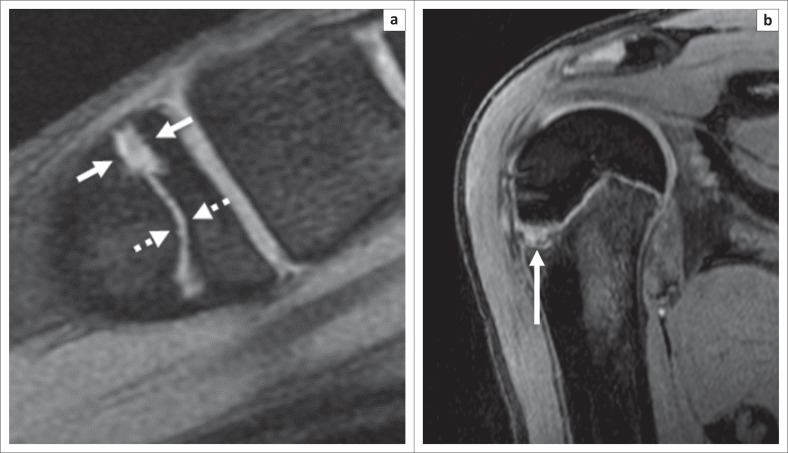
Chronic physeal stress on magnetic resonance imaging. (a) Sagittal gradient echo magnetic resonance image (959/14 ms, 20° flip angle) of the great toe in the same girl delineates abnormal widening of the dorsal physis (solid arrows) relative to the normal plantar physis (dotted arrows). The signal intensity is similar between the normal and the abnormal portions of the physis. (b) Sagittal volumetric gradient echo magnetic resonance image (13.9/5.2 ms, 30° flip angle) of the proximal right humerus of a 12-year-old male baseball player shows a focal region of abnormal widening (arrow) of the lateral aspect of the proximal humeral physis, with a configuration similar to the great toe metatarsal physis in the patient shown in Figure 2. This so-called ‘Little Leaguer’s shoulder’ is another example of physeal stress change attributed to a chronic traction injury.

Radiographs of her right foot and ankle obtained on the day of injury were initially interpreted as normal, although in retrospect, they showed subtle widening of the lateral portion of the great toe metatarsal physis (Figure 1). Because of symptoms that persisted for 6 weeks after injury, she was referred for MR imaging. The MR imaging examination revealed marrow oedema in the proximal aspect of the cuboid without a discrete fracture line, compatible with an osseous contusion (Figure 2). Focal widening of the dorsal one half of the great toe metatarsal physis with normal physeal signal intensity was noted for all sequences, but was most easily identified on gradient echo images (Figure 3). No oedema-like signal was noted in the surrounding muscles or other soft tissues.

The patient had no pain or tenderness localised to the medial aspect of the foot. However, she continued to report substantial lateral ankle and foot pain after 10 weeks of conservative management, which included physical therapy, and referral to the pain clinic, but she was lost to follow-up.

### Ethical considerations

This article followed all ethical standards for research without direct contact with human or animal subjects.

## Discussion

Physeal cartilage is the weakest structure in the developing skeleton and thus is most susceptible to injury. It is less resilient to stresses than its counterpart of adult articular cartilage, less resistant than the adjacent bone to shear and tensile forces, and two to five times weaker than surrounding ligamentous tissue.^5^ Susceptibility to physeal injury is particularly pronounced during periods of rapid growth, as occurs during pubescence.^5^

Chronic physeal stress injuries result from chronic, repetitive physeal microtrauma that can occur because of compression, distraction and/or shear forces.^3,4,5^ On imaging, chronic physeal stress injury manifests as abnormal physeal widening,^3,4^ which can often involve only a portion of the physis as a result of frequently non-uniform stress, as noted in our patient. At a histologic level, in the setting of a chronic, repetitive compression force, physeal widening results from impaired endochondral ossification possibly related to impaired metaphyseal perfusion that manifests as thickening of the hypertrophic zone with extension of physeal hypertrophic chondrocytes into the metaphysis.^4^ In the setting of a distraction force, physeal widening may be the result of persistence of hypertrophic chondrocytes that do not progress further in the process of endochondral ossification, the proliferation of chondrocytes in the hypertrophic zone^6^ and/or microscopic foci of physeal separation through the hypertrophic zone and/or at the junction of the hypertrophic zone and metaphysis.^7^

Chronic physeal stress injury as a result of both compressive and distraction-related athletic activity has been reported in association with a number of sports, which include baseball, gymnastics, soccer, running, tennis, football, basketball and rugby.^5^ Chronic physeal stress injuries can occur at several sites, including physes adjacent to the epiphyses and apophyses of the upper and lower extremities, and the apophyses of the pelvis,^3,5^ with the distribution of sites of involvement related to the specific sport. Given the predominant use of the lower extremities in soccer, it is not surprising that reported soccer-related chronic physeal injuries involve the pelvis and lower extremities.^3,5^ However, to our knowledge, no prior cases of metatarsal chronic physeal stress injury have been reported in soccer players. The condition of most athletes suffering from chronic repetitive physeal injury improves with rest, and they can return to their sport,^5^ but premature physeal closure and deformity can occur with non-compliance.^4^ Chronic physeal stress change may not be symptomatic.

**FIGURE 4:**
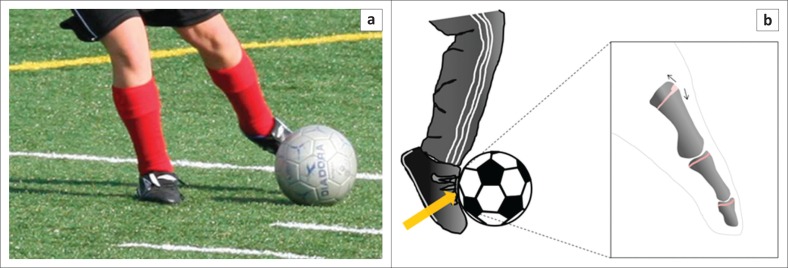
Photograph (a) and diagram (b) illustrate a possible mechanism for the dorsal physeal widening seen in our patient. We postulate that kicking of a soccer ball imparts a tension force on the dorsal aspect of the physis. Upon repetition, this can result in physeal distraction and subsequent stress injury, similar to other chronic physeal stress changes, such as the one shown in ‘Little Leaguer’s shoulder’ (Figure 3b) or elbow.

The widening of the dorsal aspect of the great toe metatarsal physis relative to the plantar aspect of the physis seen in our patient suggests that the abnormal force predominantly affects the dorsal aspect of the physis. Given the manner in which the forefoot strikes the ball during repetitive kicking in soccer, we postulate that a repetitive distraction force acts on the dorsal aspect of the physis to manifest as asymmetric chronic physeal stress changes (Figure 4). Alternatively, it is conceivable that the physeal changes may result from a chronic compressive force on the dorsal aspect of the physis which could occur during running, as there is a single case report of great toe metatarsal chronic physeal stress injury that was reported in an avid long-distance runner.^8^ However, involvement of only the great toe metatarsal in our patient and the fact that we have not encountered these changes in patients involved in other activities with substantial running lead us to favour a kicking-related and, therefore, a distraction-based mechanism.

Interestingly, chronic physeal stress injuries at other lower extremity sites have been reported in kickers.^4^ Whether the injured physis in these cases was in the planting leg and resulted from compressive force, or in the kicking leg and resulted from distraction, was not reported in either instance; therefore, it is difficult to discern whether the mechanism of injury may have been similar to the current postulated causation, but involving a different physeal location.

We were unable to determine foot dominance in this patient to correlate foot dominance with the side of injury; however, soccer training and play involves both feet; therefore, the presence or lack of similar findings in the contralateral foot would be non-contributory.^9,10^ We were also unable to ascertain how long or how frequently she had been playing soccer or what her training pattern was, although she was described as a high-level player. As distraction forces on the great toe might be present with other types of sports, additional studies to evaluate for similar chronic physeal stress changes in other child athletes would be helpful.

## Conclusion

In summary, we suggest that observed physeal widening of the great toe metatarsal in a skeletally immature soccer player may reflect asymptomatic or symptomatic chronic physeal stress changes akin to imaging findings previously described at other sites of skeletal growth in athletic children. This finding, whether associated with pain or not, should be brought to the attention of the ordering clinician by the radiologist so that activity limitation can be considered to allow the injury to heal.

## Learning points

1. Physeal widening at the base of the great toe metatarsal may reflect a chronic physeal injury that may or may not be symptomatic.
2. Similar to physeal stress injuries in other locations of the growing skeleton in children, limitation of activity to allow for healing may be needed.
